# Is It Possible to Monitor the Safest Time to Perform Secondary Surgery on Free Flaps? A Clinical Evaluation of the Tewameter^®^

**DOI:** 10.3390/medicina60081327

**Published:** 2024-08-16

**Authors:** Mahsa Bagheri, Katharina Tietz, Maria von Kohout, Paul C. Fuchs, Rolf Lefering, Jennifer L. Schiefer

**Affiliations:** 1 Clinic for Plastic and Hand Surgery, Burn Care Center, Cologne Merheim Medical Center (CMMC), University of Witten/Herdecke, 51109 Cologne, Germany; 2 Institute for Research in Operative Medicine (IFOM), Faculty of Health, University of Witten/Herdecke, 51109 Cologne, Germany

**Keywords:** free flap monitoring, free flap autonomization, sympathetic skin response (SSR), free flap secondary surgery, microsurgery

## Abstract

*Background and Objectives*: Postoperative monitoring, following free flap surgery, plays a crucial role in ensuring the survival of the flap. However, in microsurgery, not only the immediate postoperative monitoring period but also the choice of the right time for secondary surgeries is crucial for the free flap survival. There is no clear consensus concerning the right choice of timing for secondary surgery. Our aim was to evaluate transepidermal water loss (TEWL), with the objective evaluation tool Tewameter^®^ in free flap surgery to monitor flap autonomization. *Materials and Methods*: Transepidermal water loss was assessed in 20 patients with microsurgically transplanted free anterior lateral thigh (ALTP) flaps. The transplantation of the ALTP-flap and the postoperative care were administered in accordance with the standard of care of the department. Measures were taken on the free flap and normal skin at follow-ups of 1, 3, and 6 months after initial free flap transplantation. *Results*: Transepidermal water loss gradually increased to the values found in normal skin, after 6 months. The differences between the two areas demonstrated the smallest variance after 6 months, specifically in the ALTP-flap region. The largest disparities were observed between month 1 and month 6, followed by month 3 and month 6, and month 1 and month 3. *Conclusions*: Free flap autonomization and physiology are complex processes. TEWL might be a valuable parameter to monitor flap autonomization. Our results indicate that TEWL in the free flap is nearly “normal” after six months. For a clear consensus of when to perform individual secondary surgery, further studies are needed.

## 1. Introduction

Postoperative monitoring, following free flap surgery, plays a crucial role in ensuring the survival of the flap, particularly within the initial 24-hour period. This monitoring is typically carried out by plastic surgeons and trained nurses [1]. Clinical assessment involves evaluation of parameters such as color, turgor, temperature, and Doppler signals, to assess perfusion status [2,3]. Numerous studies have explored different methods for effective postoperative perfusion monitoring [3]. Thus, in the literature, a vast range of possible objective evaluation methods have been presented [2]. To name just a few, e.g., laser Doppler flowmetry, tissue spectrometry (O2C), and hyperspectral imaging have demonstrated the ability to detect earlier perfusion problems than humans through the assessment of multiple parameters [3,4]. Nevertheless, it is the clinical and poorly reproducible evaluation that remains the gold standard [2,5].

However, in microsurgery, not only is the immediate postoperative monitoring period crucial for free flap survival, but also the choice of the right time for secondary surgeries is crucial to avoid endangering the flap’s survival [6,7,8,9]. Broadly, these secondary operations can be divided into two categories: (1) surgeries for wound closure and (2) refinement surgeries [10]. Refinement surgeries include thinning procedures, such as excision of the subdermal tissue and conventional liposuction [8,9,10]. Especially in areas such as ankles, thin and pliable flaps are necessary to maintain good joint function [7]. Furthermore, fascio- and myocutaneous free flaps are shown to be independent predictors of secondary refining free flap surgeries [10].

It is crucial to choose the right time for the secondary surgery, to avoid critical ischemia, when cutting the axial blood supply [8,10]. A secondary operation would be safest when the free flap autonomization is complete. In the literature, there have been efforts to determine the safest time for secondary surgery, and, for this objective, monitoring devices have been described as well. Here, an O2C-device was described to determine flap autonomization by evaluating flap neovascularization [9].

Flap autonomization includes cutaneous flap neovascularization and parasympathetic ingrowth, despite its axial blood supply [6,9]. Differences in the anatomy of the neurovascular supply of different flaps brings further variability into the pathophysiological background of the transplanted free flaps. Rahmanian-Schwarz et al. had already suggested in an early work how to use the thermoregulatory capability as a parameter to measure sympathetic nerve innervation in transplanted tissue [11], and, thus, monitor flap autonomization.

We hypothesized that the autonomization of a free flap is mirrored in the approximation of its parasympathetic regulation, represented by the parameter of transepidermal water loss, to normal skin areas. To evaluate our hypothesis, we conducted a randomized control study with specific postoperative treatment protocols and an objective skin evaluation device to determine the skin evaporation of transplanted free flap areas.

## 2. Materials and Methods

### 2.1. Patient Cohort

The study was conducted as a randomized controlled study at Merheim Hospital, University Hospital Witten/Herdecke from 2015 to 2020. All patients who had an indication of a microsurgical transfer of an anterior–lateral thigh perforator flap for upper or lower extremity reconstruction, and met the criteria, were included.

Inclusion criteria were age over 18 years and an informed consent. Exclusion criteria were existing pregnancy, dementia, patients currently serving sentences, or patients with pre-existing psychiatric conditions. Subsequent exclusion of patients could, of course, occur upon their request, at any time.

Free flap surgery was primarily indicated for patients requiring complex reconstructive procedures when local tissue was insufficient. Indications included reconstruction of extensive soft tissue defects following trauma or oncological resections, restoration of form and function in the extremities, and cases where primary closure or skin grafts were not feasible due to the defect’s size or complexity.

Contraindications included patients with significant comorbidities that posed a high risk for anesthesia or surgery, poor recipient site vascularity, active infection at the donor or recipient site, and poor overall health status, including malnutrition or uncontrolled diabetes.

### 2.2. Anterior-Lateral-Thigh-Perforator (ALTP)-Flap

The transplantation of the ALTP-flap was performed according to clinical standards. The flap is harvested from the ventrolateral thigh region between the vastus lateralis and the rectus femoris muscles, with its blood supply derived from the femoral circumflex artery deriving from the femoral artery. For surgical planning, landmarks such as the superior iliac spine and the lateral margin of the patella are used to guide the procedure. The flap is raised, the vascular pedicle is identified and preserved, and the flap is then transplanted to the recipient site. Postoperative care involves monitoring for complications, such as ischemia, infection, and bleeding, and appropriate interventions were made to ensure optimal flap viability.

### 2.3. Postoperative Care

Postoperative care was administered in accordance with the Standard of Care (SOC) of the plastic surgery department at Merheim clinic in Cologne.

### 2.4. Tewameter^®^

Measurements were obtained during follow-up visits at 1, 3, and 6 months postoperatively, using the Tewameter^®^ device. All measurements were conducted under standardized conditions, ensuring consistency across examinations. The same examiner performed the measurements in the same room with controlled temperature conditions, to minimize variability.

The Tewameter^®^ manufactured by Courage+Khazaka electronic GmbH in Cologne was connected to the Multi Probe Adapter System. The device utilizes an “open chamber” measurement principle to determine transepidermal water loss (TEWL). This measurement can detect early skin barrier dysfunction and identify small skin damages through the probe. The principle is based on measuring the rate of water evaporation from the skin, which is captured by two pairs of sensors within a hollow cylinder (the “open chamber” measurement). The sensors measure temperature and relative humidity. These values are then converted into water vapor rate (g/h/m^2^).

This method does not affect the microclimate on the skin. The probe is placed on the skin for 30 s, allowing the calculation of the average TEWL and skin surface water loss (SSWL). The SSWL represents the skin’s water-binding capacity, post occlusion. Both the mean value and standard deviation are provided. The small-diameter, lightweight design of the measurement chamber reduces air movement within the probe and minimizes the risk of altering the skin surface [12].

### 2.5. Statistics

Statistical evaluation was conducted using IBM SPSS Statistics (version 25, IBM Corp., Armonk, NY, USA). The significance level was set at α = 0.05 for determining statistical significance. The study design employed the paired-samples *t*-test to analyze the data. In instances where complete pairs of values were not available, interpolation was used to ensure an adequate evaluation.

## 3. Results

### Study Results

The study included a total of 35 patients, of which 20 patients were able to participate in the complete follow-up measurements using the Tewameter^®^. The remaining patients were classified as drop-outs due to non-compliance or the impact of the COVID-19 pandemic, which resulted in reduced hospital visits. In five cases, a single follow-up measurement was missed, and, therefore, statistical interpolation was performed to compensate for the missing data.

The study population consisted predominantly of male individuals, accounting for 95% of the participants. Age distribution and comorbidities within the population are shown in Table 1.

All patients with diabetes had Type II diabetes; 15% of these were also obese. None of the patients had a history or manifest signs of diabetic polyneuropathy.

**Figure 1:**
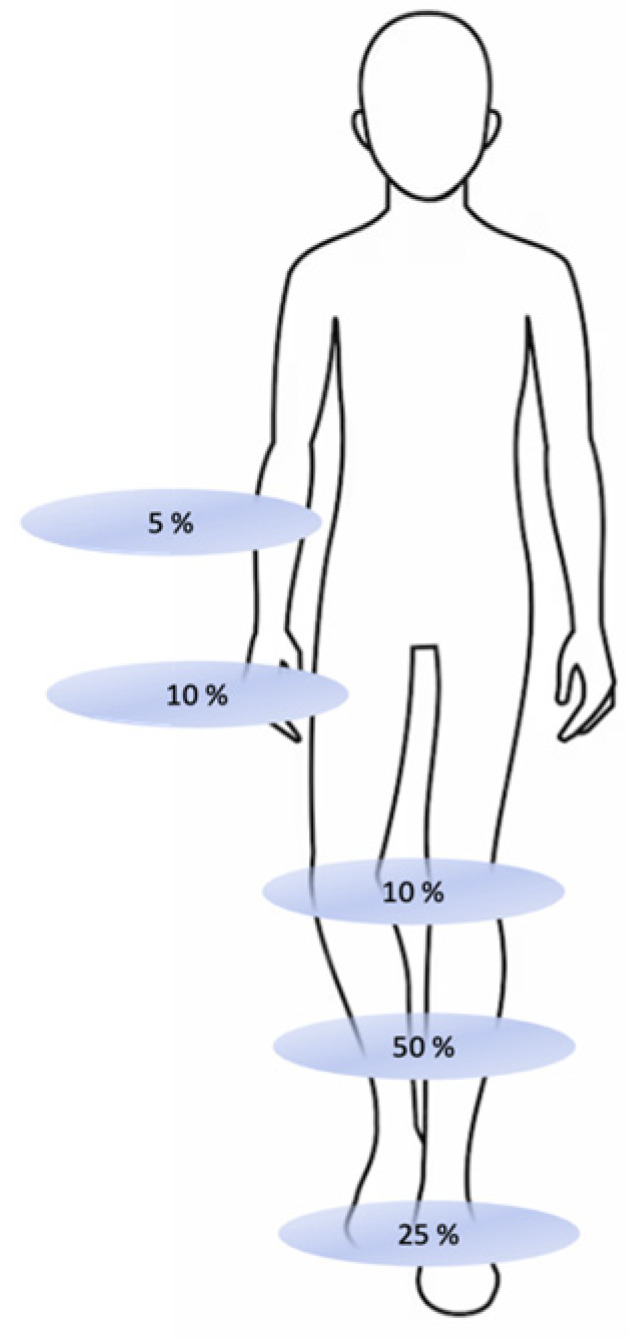
Defect localization in the study group.

The localization of the defects is shown in Figure 1, and most were in the lower extremities: 50% of the defects were localized in the lower leg, 25% in the foot, 10% in the knee, 10% in the hand, and 5% in the lower arm. 

**Figure 2:**
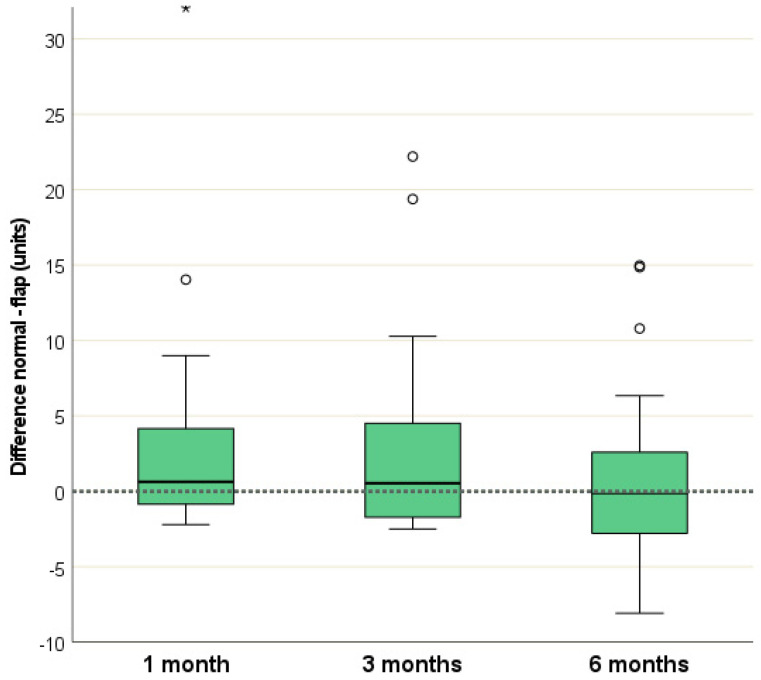
Tewameter^®^ differences (normal flap) at all follow-ups. (*) marks an extreme outlier at 1 month.

The Tewameter^®^ values obtained during follow-up assessments were consistently higher on normal skin area, compared to the ALTP-flap area. The calculated differences between the two areas were the smallest after 6 months, specifically in the ALTP-flap region, as presented in Table 2 and Figure 2. 

A comparison of the differences across the follow-up periods is displayed in Table 3. The largest disparities were observed between months 1 and 6, followed by months 3 and 6, and months 1 and 3. However, statistical analysis did not reveal any significant differences in these values.

## 4. Discussion

Recent research has produced important findings concerning immediate postoperative monitoring of freshly harvested and transplanted free flaps [13]. But there is only scarce information on methods to determine an optimal time after free flap transplantation to ensure conducting a secondary surgery is safe.

A secondary operation on free flaps is needed quite often, to ensure optimal functionality and aesthetic outcome. These secondary surgeries include procedures for wound closure or for refinement [10]. Although the need for secondary procedures on transplanted free flaps, such as debulking operations, is decreasing due to the widespread use of perforator flaps with thin subcutaneous layers (e.g., superficial artery perforator flap) [6], there is scarce information about the safest time to perform such a procedure, to ensure flap survival.

The aim of this work was to analyze a simple monitoring device that could provide information about when to perform a safe secondary operation. We hypothesized that if the thermoregulation of the transplanted free flap—represented in our study by the regulation of sweating—is intact, the microcirculatory regulation of the flap is adequate. Adequate in this case means that the microcirculatory regulation of the free flap area can provide enough blood flow besides its axial blood supply, so that the flap is not impaired when a secondary surgery for refinement is performed.

In addition to flap physiology, neural circuit pathways for body temperature hemostasis include a wide range of unique neural pathways that are not yet completely understood. Here, thermoeffector outputs include neural pathways regulating thermogenesis, shivering, and evaporative heat loss via sweating [14]. The activation of human cutaneous sympathetic nerve fibers results in active cutaneous vasodilation [14,15]. 

The sympathetic skin response (SSR) is the potential generated by sweat, in response to different stimuli [16]. It has a waveform that habituates with closely repeated stimuli and has a latency of, e.g., 1.3–1.5 s at the hand and 1.9–2.1 s at the foot. It is a somato-sympathetic reflex with a spinal, a bulbar, and a suprabulbar component with a complex pathway [17]. Its measurement uses a well-established neurophysiological method to detect impairments of the autonomic nervous system such as peripheral neuropathies with autonomic symptoms. It has been shown to be a valid instrument to describe a small section of the autonomous nervous system, in particular, the sudomotor dysfunction [17,18].

Contrary to intact skin, a transplanted free flap has been cut from afferent and efferent nerve connections, resulting in a complete sympathectomy [19]. This naturally results in impaired microcirculatory regulation. Mücke et al. showed that flap autonomization was possible even after 4 weeks, but emphasized that it is dependent on the localization of transplantation, the flap type, and the wound bed [9]. The majority of the flaps in our study were localized in the lower extremities, showing a gradual increase in the TEWL values towards that of normal skin from the follow-ups after 1 to 6 months (see Table 2). While a higher TEWL value indicates a higher level of fluid loss and could potentially suggest a disruption in the skin barrier function, in this context, the increase in TEWL could rather imply an adaptation towards the characteristics of normal skin.

Han et al. measured the SSR of 42 patients with diabetic sensorimotor polyneuropathy (DSPN) and peripheral autonomic neuropathy (PAN), as well as skin evaporation and its possible impaired skin barrier function with TEWL. They were able to confirm the hypothesis of diabetic neuropathies impairing the sudomotor function by showing the impaired skin barrier function with significantly lower TEWL values in patients with abnormal SSR values [20].These results are in line with our hypothesis that an intact autonomous nervous system is consistent with normal TEWL values. In addition to the TEWL values as an indicator for free flap autonomization, it might be interesting to examine the SSR in combination with the TEWL values during free flap autonomization. 

Our results suggest that after 6 months, the autonomization of the flaps is almost completed, and thus, a secondary operation relatively safe. Nevertheless, these results do not represent available clinical evidence: we do not present data concerning actual vascularization and concerning results after secondary surgery.

The variance of the measured differences inTEWL values is highest between the first and the sixth month. This finding indicates that the water loss of the flap tends to adapt over time, gradually aligning with the TEWL values of normal skin. Additionally, the greatest adaptation appears to take place between the third and sixth month, as indicated by the second-largest difference observed during this period. However, due to the limited number of measurements, statistical significance could not be established to support these findings conclusively.

Without having precise timing recommendations, most authors prefer to present safe options for secondary operations. Here, multistage thinning procedures, such as the direct excision of the subcutaneous tissue by elevating the flap edges only on one side of the flap, are presented to be safe, as long as the vascularity of the dermal-subdermal plexus is left intact on the counter-side [7]. Another possibility, such as conventional liposuction for flap debulking, has been shown to have good results [6], while other authors describe the risk of causing signs of ischemia even 9 months after primary flap transfer [7,21]. The individual flap design and patient characteristics would thus profit from a monitoring device, with which one can confirm operability before secondary surgery.

Similarly, in a large retrospective study of 389 patients, Kotsougiani et al. state that a secondary procedure is safe after 10 months after primary operation, without offering a monitoring possibility. Among the reasons they present for choosing this time are the elective nature of the procedure and, in most cases, the long rehabilitation processes in between [22]. In contrast, Mücke et al. described free flap autonomization in the oral cavity already after 3 months, measured with the O2C monitoring system [9]. Here, the authors present an adaptable determination of operation time. Factors influencing autonomization are flap anatomy, wound bed quality, and patient characteristics [9]. In our results, we could not detect variability concerning the flap types. However, because flaps independent of their pedicle blood supply have been reported to show late flap failure up to 3 years after free flap transfer [9], a reliable method for monitoring flap autonomization is needed.

Jaramillo del Rio et al. carried out their secondary debulking procedures of transplanted free flaps of the ankle after an average of 9.3 months (range 2–47 months) following the initial procedure. They transplanted a full thickness skin graft, grafted from the flap itself, after complete excision of the subcutaneous fat layer. All 22 procedures were successful [7]. Cayci et al. describe that most fasciocutaneous free flaps can be safely divided from their axial blood supply weeks to months after insetting. They imply that the neovascularization, the principle of peripheral vascular ingrowth, can allow for aggressive debulking surgeries 3 to 6 months after primary surgery [8], which would align with our results.

In a retrospective multicenter study by Wong et al. of 420 free tissue transfers of the lower extremity, 57% of the flaps needed secondary surgery, with the median performed after 3.6 months, and of those, 14% were for refinement surgeries. Here, they found that patients who were male and had diabetes were less likely to undergo secondary surgery and that patients with myo- and fasciocutaneous flaps were more likely to undergo refinement surgery than patients with a muscle-only flap [10].

Our hypothesis of using the thermoregulatory response in free flaps has been described already the in literature [11]. Rahmanian-Schwarz et al. suggested using the thermoregulatory response in transplanted tissue as a parameter to measure sympathetic nerve innervation in transplanted tissue, also representative of the microcirculatory capacity. Similarly to our results, they showed, in a total of 22 microsurgically transplanted ALTP and Latissimus dorsi muscle (LDM) flaps, the existence of thermoregulation, monitored by the O2C device after 6 months post transplantation [11]. 

To the best of our knowledge, in the German literature so far, there is no consensus on when to have a secondary operation on free flaps [23,24]. Because there is great variability in choosing the optimal time without adequate monitoring methods, our results can indicate a safe operation time 6 months after initial transplantation. Nevertheless, in future studies, e.g., with the simultaneous measurement of the SSR in combination with the TEWL during the healing phase of free flaps, it could be interesting to further specify the time course of the autonomization of free flaps. For this, we plan to establish further studies on free flaps to match SSR results to the TEWL measurements.

### Limitations

This study has some important limitations to acknowledge. While we maintained consistency by having a single examiner conduct all measurements, the potential for inherent inaccuracies remains. The modest size of our study population constrains the scope of statistically significant findings. To improve clinical relevance, a more extensive cohort with diverse flap types, along with data on secondary surgeries and microvasculature, is needed. Additionally, further investigations with varied designs are essential to better understand the adaptation of free flaps to normal adjacent skin. 

## 5. Conclusions

In summary, our findings suggest that transepidermal water loss (TEWL) measurements in the assessment of a transplanted free flap area could serve as valuable criteria in determining the optimal timing for a secondary surgery. Notably, our results reveal that the skin’s evaporation capacity within the free flaps closely approximates that of the normal adjacent skin after 6 months. To substantiate and validate these observations, we recommend undertaking a more extensive study involving multiple free flaps, and further variables such as the SSR, to establish statistically significant outcomes.

## Figures and Tables

**Table 1:** Baseline characteristics of the study group.

	Number of Patients (%)
GENDER	
Male	95%
Female	5%
AGE IN YEARS	
<20	5%
20–40	5%
40–60	45%
>60	45%
COMORBIDITIES	
Heart disease	40%
Lung diseases	10%
Vascular diseases	15%
Rheumatoid diseases	5%
Diabetes	20%
Obesity *	15%
Depression	5%

* BMI ≥ 35 kg/m^2^.

**Table 2:** Baseline characteristics of the Tewameter^®^ measurements of ATLP flap area vs. normal skin, *n* = 20 patients.

	F1	F3	F6	N1	N3	N6	DIFF1	DIFF3	DIFF6
mean	7.50	8.16	10.55	11.02	11.22	11.69	3.52	3.06	1.14
SD	1.84	3.32	5.42	7.43	6.99	5.23	7.90	7.01	6.35
median	7.62	7.32	8.59	9.30	10.65	11.05	0.64	0.54	−0.15

F = ALTP flap, N = normal/healthy skin area, DIFF = difference of measurements of ATLP—normal area, 1, 3, 6 = follow-ups after 1, 3 and 6 months, mean in g/h/m^2^, SD = standard deviation.

**Table 3:** Differences of the Tewameter^®^ Measurements at different follow ups.

	DIFF1–3	DIFF1–6	DIFF3–6
mean	0.45	2.37	1.92
SD	3.51	7.24	5.90
95% CI	−1.19–2.10	−1.01–5.63	−0.84–4.68
*p*	0.57	0.16	0.16

DIFF = difference in measurements between different follow-ups of ATLP—normal area, 1, 3, 6 = follow-up after 1, 3 and 6 months, mean in g/h/m^2^, SD = standard deviation, CI = confidence interval.

## Data Availability

The datasets presented in this article are not readily available because the data are part of an ongoing analysis. Requests to access the datasets should be directed to the corresponding author.
